# Does Poor Quality Schooling and/or Teacher Quality Hurt Black South African Students Enrolling for a Degree at the University of KwaZulu-Natal?

**DOI:** 10.1371/journal.pone.0153091

**Published:** 2016-04-12

**Authors:** Mike Murray

**Affiliations:** University of KwaZulu-Natal, School of Mathematics, Statistics and Computer ScienceWestville Campus, Durban, KwaZulu-Natal, South Africa; TNO, NETHERLANDS

## Abstract

**Abstract:**

Wealthy schools appoint better qualified teachers, less wealthy schools under qualified teachers. Added to this mix is a powerful teacher’s union whose policies attempt to entrench the job security of teachers in the less wealthy schools irrespective of whether they can teach their subjects or not. Can one isolate these effects from that of other socio-demographic factors that may also be affecting the performance of students when they enrol for a degree at the University of KwaZulu-Natal (UKZN)? An outcome variable that subtracts the number of courses that have been failed from the number of courses that have been passed, dividing this by the total number of years that they have spent studying for a particular degree will be used as a response variable for this paper.

**Objectives:**

The system of secondary education in South Africa is highly polarized. On the one hand, we have a group of mainly Black African students, forming about 80% of the total student population, that come from a vastly under-resourced rural or township based community. On the other hand, we have a group of predominantly White and Indian students who are able to attend a far better resourced set of private schools. Added to this mix, we have 240,000 of South Africa’s total number of 390,000 primary and secondary school teachers who belong to a powerful teacher’s union which enjoys a strong political alliance with the ruling party in South Africa. With most of their union members teaching in the less wealthy schools in South Africa, `school background’ now includes a politically motivated component that focuses on teacher self–interest rather than the education of the child. What sort of effect does school background have on the performance of students when they enter an institution of higher learning? More importantly, can one isolate the effect of school background from that of other possibly confounding factors such as gender, financial aid and the receipt of some form of residence based accommodation that will also impact on their performance while at university?

**Method:**

A total of 6,183 students enrolling for a degree at the University of KwaZulu-Natal (UKZN) over the period 2008 to 2012 were used a dataset for this study. Permission to use this dataset was given by the Teaching and Learning Office at UKZN. The database that was used for this study was obtained from the Division of Management Information (DMI) office at UKZN. The percentage based marks that students have managed to record for Mathematics, English, Biology and Accounting in their school leaving exams together with some other important but observable socio-economic factors were included in a regression model to determine how students will perform at UKZN. Socio-economic variables relating to gender, race and whether they have receivd some form of financial aid or residence based accommodation while studying at university were also included as predictor variables in our regression based model structure.

**Results and Conclusions:**

An interaction effect associated with being a Black African student who has been privileged enough to attend a quintile five school was found to be significant. A main effect associated with being able to attend a more privileged quintile 5 school however was found to be nonsignificant even after an adjustment has been made for gender, race, the receipt of some form of financial aid and residence based accommodation. Given that UKZN already has a number of bridging programs in place that target students who have come from a less privileged background, for university based policymakers, this result may help to justify the targeted selection of Black African students from the less privileged schools that is taking place. Because some of the disparity in matric performance that we are observing may also be associated with teacher competency and the protective influence of a powerful teacher’s union, this paper may also help to highlight some of the economic costs related with having under-prepared students. “A mind is a terrible thing to waste”–United Negro College Fund.

## Introduction

The poor performance of South African students in internationally benchmarked literacy and numeracy based exams has been well documented in the literature [1–3]. In a 2003 TIMSS study, grade eight learners in South Africa recorded the lowest average score amongst a total of 50 countries that took part in the test [4–6]. In a similar 2011 study, South Africa chose rather to give these benchmarked tests to their grade nine pupils with Botswana and Honduras being the only other countries to test their grade nine rather than their grade eight learners. The results from the 2011 TIMSS study showed that the mathematical knowledge of an average South African Grade 9 learner lagged behind that of an average Grade 8 learner from any one of the other participating countries by a period of at least two years. Disaggregating the results that are being recorded according to the wealth status of the schools from which they have come, however, showed that students who were able to attend a better resourced school were able to perform much better in Mathematics than those in the townships who were being forced to attend a more poorly resourced school. What sort of effect will school background have on the performance of these more poorly resourced township school based students when they enter an institution of higher learning? The regression model that we will be fitting gives one the opportunity to isolate the effect of school background from that of other confounding factors that may also be affecting their performance when enrolling for a degree at UKZN.

### School Quintiles

All public schools in South Africa are given a ranking based on the level of poverty that exists within the community in which the school is located. More specifically, this ranking is based on the average level of income, the unemployment rate and level of education within the community each of which are given a specific weighting that is determined by the Department of Education. Schools falling in the bottom 20% of this ranking (i.e. the poorest schools) are classified as being Quintile 1 schools. Schools falling within the top 20% of this ranking are said to be Quintile 5 schools. Because UKZN offers bridging programs to help supplement the knowledge base of students who have attended a quintile 1 or quintile 2 level school, identifying school background as a statistically significant effect will help justify the funding and implementation of these bridging programs that is taking place at UKZN.

### The South African Democratic Teachers Union

250,000 of South Africa’s total population of 390,000 primary and secondary school teachers belong to a powerful union called the South African Democratic Teacher’s Union (Sadtu). Being a majority member in an even more powerful grouping of trade unions which have formed an alliance with the African National Congress (ANC) who govern the country, under this protective blanket Sadtu have been allowed to promote policies that are aimed primarily at entrenching the job security of their teacher’s irrespective of whether they can teach their subjects or not. For example, in 2006, Sadtu successfully prevented initiatives by the government to weed out teachers who were classed as being `untrainable’. In 2007, they blocked a proposal to introduce school inspectors into the system. In 2011, plans to introduce competency testing for new teachers were also blocked. In 2013, plans to introduce competency testing for exam markers were also blocked. With most of their members teaching in the lower quintile township based schools of this country, under a protective Sadtu blanket, teacher absenteeism and a lack of discipline in these schools has become rife.

Having such a large membership, Sadtu has been able to forge an often militant on-site presence in almost every lower quintile school in South Africa. Other unions who wish to participate on the South African landscape are crowded out being forced to recruit their membership elsewhere primarily from the more privileged quintile 5 schools.

Based on audited 2012 figures that have been supplied to South Africa’s Public Services Bargaining Council, the National Professional Teachers Association of South Africa (NAPTOSA) forms the next biggest organisation with approximately 50,000 members. Representing to a large extent an aggregation of teacher unions from the apartheid era, under a typically white leadership their agenda has become more focused on the promotion of professional development amongst their members who (due to the militancy of SADTU) are being restricted to the more privileged quintile 5 schools of South Africa.

Consequently any significant effect that one is able to observe for school background can (in these lower quintile schools) be viewed as being largely a proxy for the poor quality of teaching that is taking place there primarily because Sadtu is pursuing an agenda based on the protection of worker’s rights rather than the empowerment of a teacher with the necessary skills to do their job properly. Including in the model an interaction effect associated with being a Black African student who has been privileged enough to attend a quintile five school, and being able to observe a significant interaction effect but a non-significant main effect would allow one to isolate the effect of being forced to attend a “poor quality” school as a Black African students from that of the other race groups who may also (by circumstances) be forced to attend a ‘poorer-quality” school.

### Response variable

A student enrolling for a three or four year degree at UKZN typically has to complete a total of 16 half-credit courses every year in order to graduate in the minimum period of time. Completion of a particular course requires a pass mark of 50% or more for that subject. Thus a student who has enrolled for a 4-year degree will have to pass a total of *16x4 = 64* half credit courses before being able to graduate with that degree. A student who has enrolled for a 3-year degree will have to pass a total of *16x3 = 48* half credit courses before being able to graduate with that degree. One could of course consider using a weighted average mark for all their courses as a response variable for this paper. Graduation however is based on the actual number of courses that have been passed rather than on a weighted average mark for an entire degree. For this reason a response variable that focuses on graduation and/or the proportion of courses that have been passed along the way was felt to be a more appropriate response variable to use for this paper. Using eventual graduation as a response variable however would result in us having to omit from our dataset all those students who are still busy with their studies when the data collection period ends. For this reason a trade-off between the total number of courses that have been passed and the number that have been failed was used as a measure of performance with the following correction being made for the total number of years that a student has been taking in order to complete their degree

$$Y_{i}=\frac{Total\;number\;of\;courses\;passed\;by\;student\;i-\;Total\;number\;of\;courses\;failed\;by\;student\;i\;}{Total\;number\;of\;years\;spent\;at\;university\;by\;student\;i}$$

Essentially *Y*_*i*_ represents a per annum based `rate of progress’ with positive valued outcomes for this response variable indicating better performers.

One could also have considered using a more simplified version of the above response variable, namely

$$\tilde{Y_{i}}=\frac{Total\;number\;of\;courses\;passed\;by\;student\;i\;}{Total\;number\;of\;courses\;taken\;by\;student\;i}$$

but $\tilde{Y_{i}}$ does not penalize the performance of a student who has been able spread their courses over a longer period of time, thereby giving them a greater capacity to pass a higher proportion of these courses but at the same time taking a longer period of time to complete their degree. Because a subsidy from the South African government is only being paid to the university based on the number of students who are able to complete their degree within a minimum period of time, it was felt, from a subsidy earning point of view, that *Y*_*i*_ would serve as a more appropriate response variable to use for this study.

### School leaving subjects

The percentage based marks that a student obtains for Mathematics, English, Biology and Accounting when they leave school will be used as possible predictors for university based performance at UKZN. These marks arise from exam papers that have been set and marked at a national level. Consequently any mark that a student obtains reflects a performance measure relative to the rest of South Africa rather than a mark that can be subjectively manipulated by the school from which they have come. These four subjects were chosen specifically because they have been identified as important drivers for many of the disciplines that are being taught at UKZN [7–10].

Many studies [11–19] have confirmed a link between academic achievement at a university and a variety of school based and socio-demographic variables ranging from the type of grade that is being recorded, the type of school that is being attended, some sort of measure of socio-economic deprivation or neighbourhood participation and the sex and race of the student. For example, in an important United Kingdom based study, Thiele et al [20] found that a student from a more deprived area did not perform as well as a student from a more affluent area. Asian and Black students did not perform as well as White students. Female students performed better than their male counterparts in the more language oriented disciplines. Contrary to expectation but in support of other studies that have been done [21–24] they also found that students who had the opportunity to attend a more privileged fee-paying independent school were not performing as well at university as students who had come from a less privileged non-fee paying state school despite these students having entered university with a higher set of school grades. An “Equal Opportunity Study” report conducted by Coleman et al. in the United States [25], however, has found that the qualitative and quantitative characteristics of the school do not always have a significant effect on the academic success of student at a university level. Instead, they found that academic performance was being determined primarily by the socio-economic characteristics that are peculiar to that family and to social and environmental factors that are often unrelated to the type of school they have been able to attend.

In a South African context, however, the system of apartheid that has been put in place has forced people with similar socio-economic backgrounds to live together in townships and rural settlements. The school they are being forced to attend will therefore more than likely reflect very closely the socio-economic characteristics of each family and thus the community as a whole. Because of this dysfunctional schooling system and poor teacher quality the marks that student’s obtain for Mathematics, English, Biology and Accounting may not necessarily reflect their true academic potential. Consequently, their performance in these subjects may (or may not) have a significant effect on their level of performance at UKZN.

### University specific and other socio-economic factors

Demographic factors that are expected to affect the performance of students at UKZN have also been included in the model building process. Dummy variables were used to incorporate the effects of gender and race on university performance in the model. Several studies have found that race and gender often play an important role in determining performance at a university. For example, male students tend to outperform females in the engineering, economics and business oriented disciplines [26–27]. In a UKZN context, females tend to perform better than males in the more language oriented disciplines [28]. Siegfried and Fels [29] have observed that whereas there appears to be little difference between the performance of males and females at an elementary (primary) school level, a definite gap begins to emerge during their high school years which then persists at university.

Research conducted by the Higher Education Funding Council for England [30] has found that race appears to have a greater effect on student performance at university than gender, one’s socio-economic background or the type of school that has been attended. Given South Africa’s racially segregated past, however, one would expect to see race playing an important role in determining how a student will perform at university. This effect due to race may however be confounded by the socio-economic status of the area from which a student has come and the type of school they have been able to attend.

Thiele et al [20] have found that school grades, school type, socio-economic deprivation, neighbourhood participation, sex all have an important effect on academic achievement at a British university. Students from the more deprived areas do not perform as well as students from the more affluent areas. Likewise, Asian and black students do not perform as well as white students and female students tended to perform better than their male counterparts.

Focusing on university specific factors that may be affecting their performance at UKZN, dummy variables were also used to indicate whether they had received some form of financial aid or some form of residence based accommodation whilst studying for their degree. Having financial security and a place to stay should impact positively on their performance at UKZN. Their age of enrolment into UKZN (measured in years) was also considered as a predictor variable in the model. One would expect older students to perform better having had a longer period of time to develop the resources that are necessary to adjust to a new learning environment. Studies [31–33] however indicate that age does not necessarily play a significant role in determining how one performs at a university and therefore age is not controlled for in this paper.

### Statistical analysis

36,804 students enrolled for an undergraduate degree at UKZN over the period 2008 to 2012. Because Mathematics, English, Biology and Accounting have been identified as being important drivers [34–40] for many of the disciplines that are being taught at a university in South Africa, only those students who have been able to do all of these subjects were included in this study. This resulted in a total of 6,183 student records being collected. The mean percentage based marks that appear in Table 1 suggest (for this very specific cohort of students) that the marks that are being recorded for Mathematics and Accounting are slightly lower than those for English and Biology.

**Table 1:** Mean percentage based marks (and standard deviations) for our reflective variables.

Subject (n = 6183 students)	Mean	Standard Deviations	Min	Max
Maths	68.974	16.21	2	100
English	74.404	8.73	34	99
Biology	72.190	11.01	20	100
Accounting	70.752	14.84	21	100

Table 2 indicates that a majority of these students (many of whom are Black African) come from a quintile 5 school. A Chi-square test for independence of the row and column factors in Table 2 proved to be highly significant (*χ*^2^ = 155.15, p-value≈0.001). A significant number of Black African students, however, are also being drawn from one of the poorer quintile level 1–4 schools. Because the policies of Sadtu play such an important role in determining the quality of teaching that is taking place in these lower quintile schools, an interaction term between these two factors needs to be included so that we can isolate the performance of Black African student who has been able to attend a quintile 5 school from one who has not.

**Table 2:** Student demographics associated with those who have been able to do Mathematics, English, Biology and Accounting as a school leaving subject.

	Black African(%)	Non Black African(%)	Total
Quintile 5 school	1057 (17.1%)	3469(56.1%)	4526
Quintile1-4 school	652(10.5%)	1005(16.3%)	1657
Total	1709(27.6%)	4474(72.4%)	6183

**Fig 1:**
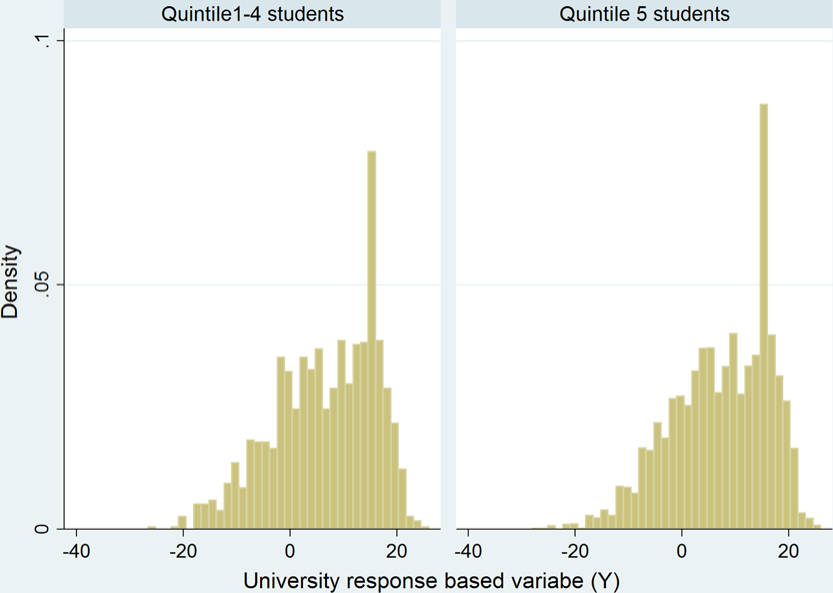
A comparison of the university based response variable (Y) for Quintile 5 and Quintile 1–4 schools.

In order to determine how they have performed at university the total number of courses that each student has passed, the total number of courses they have failed and the total number of years they have been registered for were collected along with some other background variables that we thought may affect their performance at university. These background variables included a 0/1 indicator variable that was set equal to 1 if they had received some form of financial aid and another 0/1 indicator variable that was set equal to 1 if they had been given some residence based accommodation during their university based studies. Table 3 indicates that the Quintile 5 students have performed better with respect to our chosen response variable *Y*_*i*_. Typically students completing 16 courses every year will complete their degree in the minimum prescribed period of time. Some of the better performing students however may choose to complete more than 16 courses on a per annum basis which will account for some of the positive skewness that we see in the plots that appear in Fig 1.

**Table 3:** A statistical summary of the university based response variable (Y) by school quintile.

Response variable (Y)	Number of students	Mean	Standard Deviation
**Quintile 5 school**	4526	7.375	9.122
**Quintile 1–4 school**	1657	6.505	9.426

Results obtained from fitting a regression model linking performance at UKZN to their matric subject marks, their race and gender, whether they have attended a quintile 5 school and an interaction term denoting whether as a Black African child they have been able to attend a quintile 5 school are given in Table 4.

**Table 4:** Parameter estimates resulting from fitting a regression model to our chosen university based performance measure Y.

Covariates	Estimate(*denotes significant at 5% level)	Standard Error	[95% Confidence Interval
Quintile5	-0.035(b)	0.305	[-0.632; 0.562]
Quintile5 *African	1.772*(a)	0.526	[0.740; 2.804]
English	0.027	0.018	[-0.008; 0.063]
Maths	0.007	0.009	[-0.009; 0.024]
Accounting	0.093*	0.011	[0.071; 0.114]
Biology	0.147*	0.016	[0.116; 0.178]
Male	-1.605*(c)	0.249	[-2.095; -0.115]
African	-1.008*(d)	0.497	[-1.982; -0.034]
Financial Aid	0.00098*(e)	0.00082	[0.00082; 0.00115]
Residence	-0.242	0.433	[-1.090; 0 .607]
Constant	-12.845*	1.158	[-15.116; -10.574]

### School quintile

The path based estimate that has been labeled (a) in Table 4 indicates that Black African students who attend a more privileged quintile 5 school are performing better than their otherwise identical Black African counterparts who have been forced by attend a lower quintile school even after an adjustment has been made for gender, race, the receipt of some form of financial aid and residence based accommodation. Although this result is to be expected given the better resourcing that occurs in these quintile 5 schools, for UKZN based policymakers, this result is important because it helps to support the targeted selection into bridging programs of students from the less privileged schools that is taking place.

The direct effect of school quintile 5 on university performance (labelled (b) in Table 4) is not significant. If it were significant then this would be an effect arising from a model where an adjustment has already been made for being a Black African student who has also been able to attend a quintile 5 school. Thus it represents an effect on Y, associated with being a White or Indian student who has also been able to attend a much better resourced quintile 5 school. Thus, being a Black African student who has been able to attend a more privileged quintile 5 school produces a better outcome at UKZN whereas being a White or Indian student who has also been able to attend a much better resourced quintile 5 school does not.

### Gender, Race and other potential factors

The path based estimate that has been labeled (c) in Table 4 indicates, for this very specific cohort of students, that females tend to perform better at university than males. Having made an adjustment for Black African students who have been able to attend a quintile 5 school, other Black African students do not perform as well at university (see the effect labelled (d) in Table 4). Focussing on their school leaving subjects, the results that they obtain for English and Mathematics do not seem to have a significant effect on their performance at UKZN. Their results for Accounting and Biology however do seem to have a significant effect on their performance at UKZN.

### Financial aid

The response variable that we have called Financial Aid in Table 4 is a 0/1 dummy variable that we have set equal to 1 if the student has been given some form of financial aid during their university based studies. The path based estimate that has been labelled (e) in Table 4 would suggest that having access to some form of financial aid should help students with this type of aid to perform better at university than an otherwise identical student counterpart who does not receive this aid. Because one is only eligible to receive this type of financial aid if one’s family based income is below a certain threshold, this variable can also be viewed as providing a proxy for some sort of `socio-economic opportunity index’ from which this student has come.

### A sample selection caveat

It should be noted that these results hold true for a very specific population of students, namely those who have been able to take Mathematics, English, Biology and Accounting at school and who have enrolled at UKZN during the period 2008 to 2012. Because our sample comprises 6183 students out of a total of 36804 who actually registered for a degree at UKZN over the data collection period, the performance at UKZN of the other students (who have not been able to take all of these 4 subjects at school) may differ from what we have have been observing for this cohort of students.

## Supporting Information

S1 Dataset Dataset containing school leaving marks and university specific demographic factors. (DTA)
